# Expression Pattern of Peroxisome Proliferator-Activated Receptors in Rat Hippocampus following Cerebral Ischemia and Reperfusion Injury

**DOI:** 10.1155/2012/596394

**Published:** 2012-12-09

**Authors:** Hong Wang, Rong Jiang, Qin He, Yunmei Zhang, Yanli Zhang, Yong Li, Ruichun Zhuang, Ying Luo, Yu Li, Jinyuan Wan, Yong Tang, Huarong Yu, Qingsong Jiang, Junqing Yang

**Affiliations:** ^1^The College of Pharmacy, Chongqing Medical University, Chongqing 400010, China; ^2^The College of Basic Medicine, Chongqing Medical University, Chongqing 400010, China; ^3^The First Affiliated Hospital, Chongqing Medical University, Chongqing 400010, China; ^4^School of Nursing, Chongqing Medical University, Chongqing 400010, China; ^5^Department of Pharmacology, Chongqing Medical University, Chongqing 400010, China

## Abstract

The present study was designed to investigate the pattern of time-dependent expression of peroxisome proliferator-activated receptors (PPAR**α**, **β**, and **γ**) after global cerebral ischemia and reperfusion (I/R) damage in the rat hippocampus. Male Sprague Dawley (SD) rats were subjected to global cerebral I/R. The rat hippocampi were isolated to detect the expression of PPARs mRNA and protein levels at 30 min–30 d after I/R by RT-PCR and Western blot analysis, respectively. The expression levels of PPARs mRNA and protein in the rat hippocampus significantly increased and peaked at 24 h for PPAR**α** and **γ** (at 48 h for PPAR**β**) after I/R, then gradually decreased, and finally approached control levels on d 30. The present results suggest that global cerebral I/R can cause obvious increases of hippocampal PPARs mRNA and protein expression within 15 d after I/R. These findings may help to guide the experimental and clinical therapeutic use of PPARs agonists against brain injury.

## 1. Introduction

Cerebral ischemic injury is the second leading cause of death and a common cause of disability worldwide. Cerebral ischemia can be divided into two groups: global cerebral ischemia and focal cerebral ischemia. The major manifestation of cerebral ischemia is a temporal or permanent reduction in cerebral blood flow, which is insufficient to meet the metabolic or functional demand of the central nervous system (CNS). There was no reperfusion after permanent occlusion of artery. Following transient ischemia or treatment of thrombolysis, reperfusion inevitably occurs. Although the reperfusion is helpful for restoring the supply of blood and oxygen to the CNS, a growing body of evidence supports the viewpoint that reperfusion may exacerbate the injury initially resulting from ischemia and is referred to as cerebral ischemia and reperfusion (I/R) injury. Cerebral I/R can cause serious neuronal injury and death, which can further lead to learning and memory impairment and neurodegeneration.

The pyramidal neurons of the hippocampal CA1 region are essential for spatial learning and memory functions. When suffering from cerebral ischemia insult, the hippocampal pyramidal neurons are the most vulnerable to the reduction of blood supply to the brain, and cell death occurs days after the initial ischemic insult, a phenomenon termed “delayed neuronal death” [1]. Currently, the mechanisms of neuronal injury and death induced by cerebral I/R are not completely known, and therefore an effective therapy for ischemic cerebral damage has remained elusive. Recently, much evidence has emerged indicating that the peroxisome proliferator-activated receptors (PPARs) are promising candidates as pharmacological targets for cerebral ischemic damage [2, 3].

PPARs, which are ligand-activated transcription factors belonging to the nuclear receptor superfamily, have three isoforms, *α*, *β*/*δ*, and *γ*. Depending on differences in their tissue distribution and transcription of target genes, the three PPAR subtypes show distinct physiological and pharmacological functions. PPARs are expressed in neurons of numerous brain regions, especially in the hippocampus [4–6]. It is well known that CNS inflammation and oxidative stress are involved in pathophysiological mechanisms of cerebral damage. In addition to regulating metabolism, activation of PPARs results in anti-inflammatory and antioxidative effects by transrepression of transcription factors (e.g., NF*κ*B) [7]. Recent studies have shown that activation of PPARs is helpful in regulating neuronal death in ischemic brain injury and neurodegenerative diseases.

Prophylactic administration of gemfibrozil, a PPAR*α* selective agonist, was shown to significantly reduce the infarct area and improve cortical blood flow in mice with permanent middle cerebral artery occlusion [8]. Collino et al. also reported that the PPAR*α* agonist WY14643 can significantly depress cerebral oxidative stress and inflammatory responses induced by transient cerebral ischemia reperfusion and that the effect of WY14643 can be abolished by the administration of MK886 (an antagonist of PPAR*α*) [9].

PPAR*β*-null mice exhibit a significant increase in the infarct size in a model of focal cerebral ischemia by middle cerebral artery occlusion (MCAO) [10, 11]. Intracerebroventricular administration of PPAR*β* agonist L-165041 or GW501516 can significantly attenuate the ischemic brain infarct size 24 h after a transient middle cerebral artery occlusion/reperfusion [12]. Another PPAR**β** agonist, GW0742, has also demonstrated an obvious protective effect against cerebral damage induced by global cerebral I/R in rats [13]. Pereira, et al. found that L-796449, a synthetic nonthiazolidinedione PPAR*γ* agonist, significantly decreases the infarct size induced by permanent MCAO and improves neurological scores and that this protection is related to inhibition of MCAO-induced expression of an inflammatory mediator [14].

Intracerebroventricular administration of pioglitazone, an agonist of the PPAR*γ*, over a 5-day period before and 2 days after MCAO (occlusion of the MCA for 90 min with subsequent reperfusion) was found to reduce the infarct size and the expression of tumor necrosis factor *α* (TNF-*α*) and COX-2 [15]. In another study, 129/SV mice were subjected to 30 min filamentous MCAO followed by reperfusion. Pioglitazone given acutely after transient brain ischemia/reperfusion reduced the lesion size. However, analysis at 6 weeks after MCAO/reperfusion indicated that pioglitazone no longer yielded an effect on lesion size [16]. Pioglitazone can also reduce delayed neuronal damage induced by common carotid artery occlusion I/R [17]. Additionally, rosiglitazone (RGZ) (5 mg/kg) intraperitoneally injected at 24 and 48 h after MCA embolization induced by placing a preformed clot into the middle cerebral artery can reduce ischemic injury and improve neurological outcome [18]. 

These previous studies indicated that activation of the PPAR signaling pathway may have significant protective effects on cerebral damage by I/R, and PPAR agonists may be candidates for ischemic brain injury. This protective effect also depends on the levels of PPAR cofactors [19]. However, the expression of hippocampal PPARs (PPAR*α*, *β*, and *γ*) after global and focal cerebral I/R injury has not been characterized.

There are two types of rodent models for clinical cerebral I/R: experimental global ischemia and focal ischemia models. An experimental rat model of global ischemia is established by bilateral common carotid artery occlusions combined with systemic hypotension. This model produces markedly reduced forebrain blood flow and results in a reversible high-grade forebrain ischemia change within selectively vulnerable structures, including the CA1 pyramidal neurons of the hippocampus, caudoputamen, and neocortex. The model is suitable for studying phospholipids and energy metabolism in ischemia and recently has been used for the evaluation of neurotransmitter metabolism, histopathology, spatial learning and memory, and the protective effects of mild cerebral hypothermia and drugs [20]. In order to explore the therapeutic benefits of PPARs agonists for the treatment of ischemic cerebral injury, this study was designed to observe the characteristics of time-dependent expression of PPARs (PPAR*α*, *β*, and *γ*) in a rat model of global cerebral I/R. 

## 2. Materials and Methods

### 2.1. Reagents

The following reagents were obtained commercially: RNAlater RNA stabilization reagent (Qiagen, Germany); BIOZOL total RNA extraction kit (BioFlux, Japan); ReverTra Ace-*α* reverse transcription kit (TOYOBO, Japan); mouse anti-rat PPAR*α*, *β*, and *γ* monoclonal antibodies (1 : 1000) (Abcam, England); mouse anti-rat superoxide dismutase 2 (SOD2) and mitochondrial uncoupling protein 2 (UCP2) monoclonal antibodies (1 : 1000) (Beijing Biosynthesis Biotechnology, LTD, China); BCA (bicinchoninic acid) protein detection kit (Shanghai Biocolors, China); ECL chemiluminescence detection kit (Pierce Biotech., USA); Taq DNA Polymerase (Promega, USA).

### 2.2. Animals and Experimental Protocol

Male Sprague Dawley rats (*n* = 117, 200–250 g and 8 weeks old) were purchased from the Laboratory Animal Center of Chongqing Medical University, Chongqing, China. They were housed in standard conditions of 25 ± 1°C, 50 ± 2% humidity, with 12 h light/dark cycles (light from 8:00–20:00). All experimental procedures were approved by the Chongqing Medical University Institutional Animal Ethics Committee. Rats were divided into one control group receiving a sham operation (*n* = 20) or eight experimental groups all receiving global cerebral ischemia followed by reperfusion for 30 min (*n* = 11), 2 h (*n* = 11), 6 h (*n* = 11), 24 h (*n* = 11), 48 h (*n* = 11), 7 d (*n* = 20), 15 d (*n* = 11), or 30 d (*n* = 11).

### 2.3. Preparation of Rat Global Cerebral I/R Model

Rats were anesthetized with 4% chloral hydrate (1 mL/100 g, ip) and fixed in a supine position. One side of the common vena jugularis and the bilateral common carotid arteries were exposed. Blood (2.4 mL/kg) was taken from the common vena jugularis, and the bilateral carotid arteries were occluded using artery clamps for 20 min. After ischemia for 20 min, the artery clamps were removed followed by blood retransfusion and different periods of reperfusion. Rats in the sham operation group were subjected to the same operation as above, except for the bilateral carotid artery occlusion and hemospasia from the common vena jugularis.

### 2.4. Morris Water Maze Test

Considering the trauma induced by the operation, only rats in the reperfusion for 7 d after global cerebral ischemia group (*n* = 9) were selected to observe changes of learning and memory in a behavior test along with rats in the sham-operated group (*n* = 9). Rat spatial learning and memory were tested using a DMS-2 Morris water maze (Institute of Materia Medica, Chinese Academy of Medical Sciences, Beijing, China) with a diameter of 1.5 meter, height of 0.5 meter, water depth of 0.4 meter, and temperature of 24-25°C. Rats were allowed to learn how to navigate the maze for four days before spatial memory was tested as previously reported [13]. 

### 2.5. Pathomorphological Observation

Three rats were selected for histopathological observation at each time point of 30 min, 2 h, 6 h, 24 h, 48 h, 7 d, 15 d, and 30 d after global cerebral ischemia (total of 24 rats). Rats in each group were anesthetized with 4% chloral hydrate (1 mL/100 g, ip) and transcardially perfused with 100 mL of 0.9% saline containing heparin (250 U) followed by 200 mL of fixing solution containing 3.5% formaldehyde and 0.1 M phosphate buffer (pH 7.2). The brain tissue was isolated and cut into coronal sections of 5 *μ*m in thickness. The sections were stained with hematoxylin and eosin (H&E). The histomorphology of neurons in each rat hippocampus was observed by light microscopy. For assessment of cell counts from H&E-stained sections, 10 consecutive high-power fields were sampled from the dorsal hippocampal CA1 subfield. Counts of intact neurons were performed from the ischemic and sham brains using a microscope at 400x magnification, and the extent of cell death was estimated. 

### 2.6. RT-PCR Analysis of *PPARs*, *SOD2,* and *UCP2* mRNA

Eight sham operation rats and eight rats at each time point of 30 min, 2 h, 6 h, 24 h, 48 h, 7 d, 15 d, and 30 d after global cerebral ischemia were sacrificed (total of 72 rats.) Brains were removed, and the hippocampi were separated for analysis of mRNA and protein expression. Thirty-six of the rat hippocampi were used for expression analysis of PPARs (*n* = 4 per time point) and the other thirty-six for SOD2 and UCP2 (*n* = 4 per time point) by RT-PCR. Total RNA was extracted from the cerebral hippocampus of rats using BIOZOL reagents according to the manufacturer's directions of the Total RNA Extraction Kit. Sequences of the primers used for RT-PCR amplification and lengths of the products (in brackets) were as follows: PPAR*α* (407 bp) forward 5′-ACGATGCTGTCCTCCTTGATG-3′ and reverse 5′-GCGTCTGACTCGGTCTTCTTG-3′; PPAR*β* (212 bp) forward 5′-GCCGCCCTACAACGAGATCA-3′ and reverse 5′-CCACCAGCAGTCCGTCTTTGT-3′; PPAR*γ* (143 bp) forward 5′-CCCTTTACCACGGTTGATTTCTC-3′ and reverse 5′-GCAGGCTCTACTTTGATCGCACT-3′; SOD2 (500 bp) forward 5′-GGCACCTTTCTCAGTAGCGG-3′ and reverse 5′-CTAAGGGACCCAGACCCAAC-3′; UCP2 (391 bp) forward 5′-CTACAAGACCATTGCACGA-3′ and reverse 5′-CTCATAGGTGACAAACATTA-3′; *β*-actin (540 bp) forward 5′-GTGGGGCGCCCCAGGCACCA-3′ and reverse 5′-CTTCCTTAATGTCACGCACGATTTC-3′.

Two-step RT-PCR was carried out according to the system manual. The total reaction volume for RT was 20 *μ*L, including 4 *μ*L 5× RT buffer, 2 *μ*L of 10 mM dNTP, 1 *μ*L of 10 U/*μ*L RNase inhibitor, 1 *μ*L of 10 pmol/*μ*L oligo (dT), 1 *μ*g of RNA template, 1 *μ*L of ReverTra Ace-*α*, and 10 *μ*L of RNase-free H_2_O. The RT conditions were 30°C for 10 min, 42°C for 20 min, 99°C for 5 min, and 4°C for 5 min. The total reaction volume for PCR was 25 *μ*L, including 5.0 *μ*L of cDNA, 0.5 *μ*L of 10 mM dNTP mixture, 2.0 *μ*L of 25 mM MgCl_2_, 2.5 *μ*L of 10× PCR buffer, 0.5 *μ*L of forward/reverse primers, 0.125 *μ*L of 2.5 U/*μ*L Taq DNA Polymerase, and 13.875 *μ*L of sterile double-distilled water. The PCR conditions were 94.0°C for 4 min, 35 cycles of 94.0°C for 15 s, 55.2°C for PPAR*α* (57.0°C for *PPAR*β**, 53.1°C for *PPAR*γ**, and 55°C for *SOD2 *and *UCP2*) for 15 s, and 72.0°C for 40 s, followed by extension at 72.0°C for 5 min.

The PCR products were visualized after electrophoresis on a 1% low melt point agarose gel and stained with 0.5 *μ*g/mL ethidium bromide for 10 min. The integrated gray values of the product bands were measured using a gel imaging and analysis system (Bio-Rad, Hercules, CA, USA). The *PPAR*, *SOD2*, and *UCP2 *mRNA levels were calculated as ratios of the corresponding **β*-actin* mRNA level (*PPARs*, *SOD2*, and *UCP2*/**β*-actin*).

### 2.7. Western Blot Analysis of PPARs Protein Expression

Proteins from the hippocampal tissues were extracted by adding 0.5 mL of tissue lysate solution and then by centrifuging at 12,000 ×g for 5 min at 4°C. The supernatant was collected for Western blotting analysis. The protein concentration was detected by the BCA method according to the manufacturer's directions of the protein detection kit. The proteins (50 *μ*g per sample) were separated by 10% (weight/volume) sodium dodecyl sulfate polyacrylamide gel electropheresis (SDS-PAGE) and then transferred to a polyvinylidene difluoride (PVDF) membrane. The membrane was blocked for 1.0 h in PBS containing 5% fat-free milk (weight/volume) and 0.2% Tween 20 (vol/vol). The blot was incubated overnight at 4°C with antibodies either to PPARs, SOD2, UCP2, or *β*-actin at 1 : 1000 dilution, followed by incubation for 1 h at 37°C with a secondary antibody (1 : 1000). Immunoreactive bands of PPARs, SOD2, UCP2, and *β*-actin were visualized with an ECL chemiluminescence detection kit, and the optical density bands were detected using a gel imaging and analysis system (Bio-Rad). The protein levels of PPARs, SOD2, and UCP2 were calculated as ratios of the corresponding *β*-actin protein level.

### 2.8. Statistical Analysis

All data were calculated as mean ± SD. The differences between groups were evaluated using one-way analysis of variance (ANOVA), followed by Dunnett's *t*-test analysis to compare between the I/R-treated group and sham operation group with the SPSS12.0 software package. Results were considered statistical significantly with *P* values <0.05. 

## 3. Results

### 3.1. Morphological Changes of Rat Hippocampus

**Figure 1:**
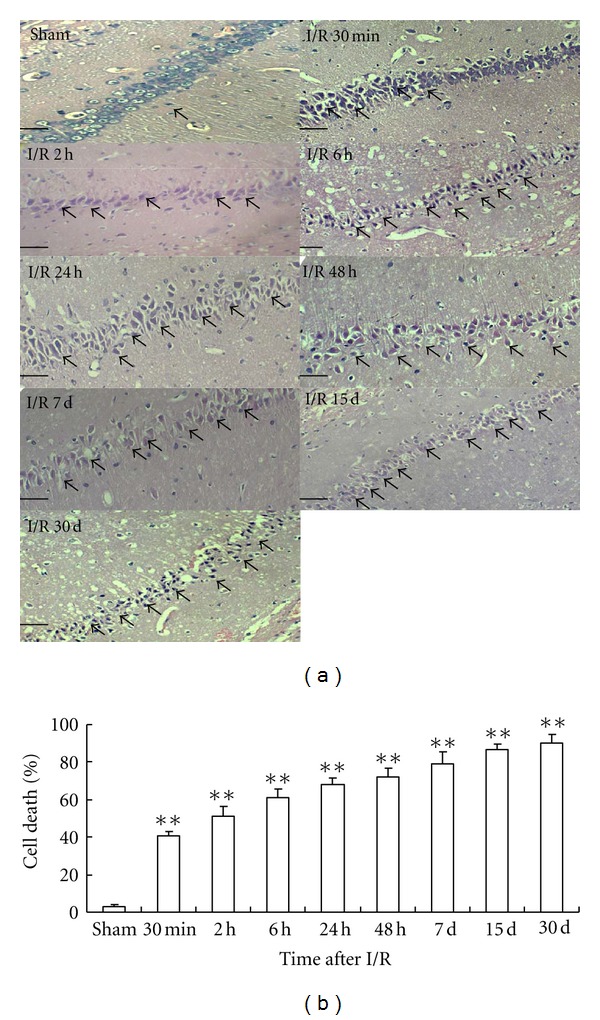
Morphological change of rat hippocampal neurons induced by I/R. (a) Representative pictures of H&E stained CA1 section, 400x. Scale bars = 50 *μ*m. (b) Group data showing the cell death rate. **P < 0.01 compared with vehicle sham group (*n* = 3).

Rat hippocampal neurons in the sham operation group were closely arranged and well structured, with clear and intact cellular form and structure. Meanwhile, neuronal karyopyknosis and reduction in the number of neurons were observed in the hippocampal CA1 subfield in I/R rats (Figures 1(a) and 1(b)).

### 3.2. Changes in Spatial Learning and Memory in Rats

Compared with that in the sham operation group, the time for rats to learn to navigate the maze from d 2 to d 4 significantly increased in the 7 d reperfusion group. For spatial memory function in rats, the time taken to find the platform was significantly longer in the 7 d reperfusion group compared with the sham operation group (Table 1). 

### 3.3. Expression of PPARs, SOD2, and UCP2 mRNA and Protein

**Figure 2:**
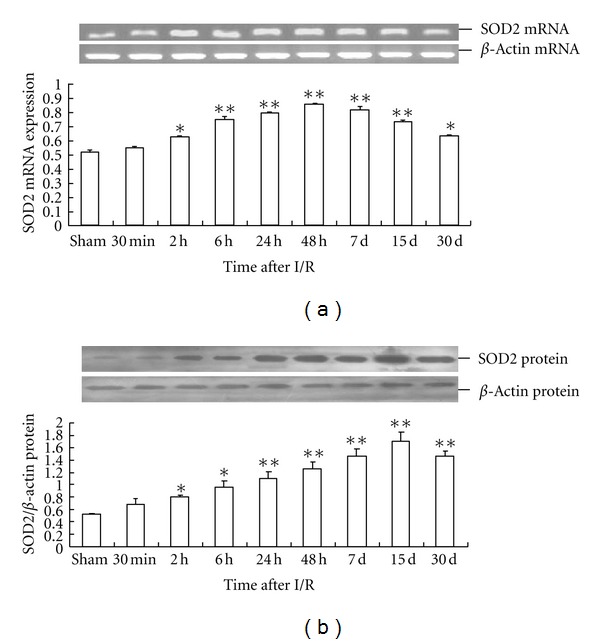
Time-dependent expression of SOD2 in global cerebral I/R rat hippocampus (*n* = 4). (a) The relative mRNA level of *SOD2* was normalized to endogenous *β*-actin mRNA for each sample. (b) The relative protein level of *SOD2* was normalized to endogenous *β*-actin protein for each sample. Dates are expressed as mean ± SD of four individual experiments. **P < 0.01 compared with sham group; *P < 0.05 compared with sham group.

Global cerebral I/R significantly increased the expression of *SOD2* mRNA in the rat hippocampus between 2 h and 15 d after I/R, with the peak expression at 48 h. The change of SOD2 protein levels in the rat hippocampus was similar to that of *SOD2 *mRNA expression, except that the peak time of expression was at 15 d (Figures 2(a) and 2(b)).

**Figure 3:**
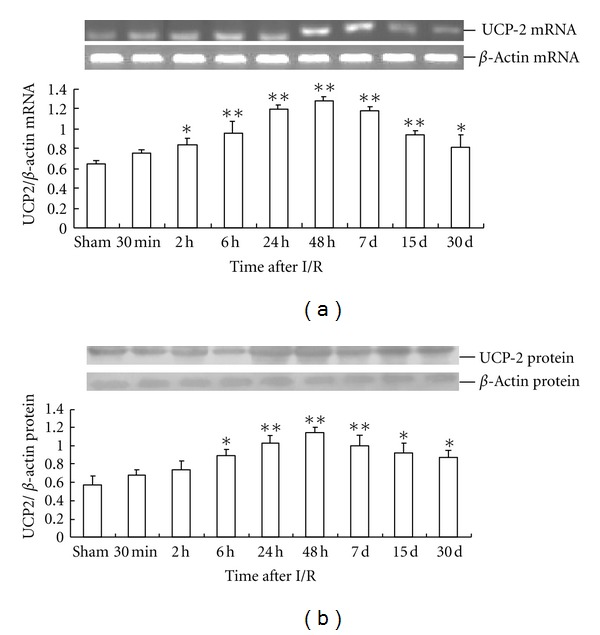
Time-dependent expression of UCP2 in global cerebral I/R rat hippocampus (*n* = 4). (a) The relative mRNA level of UCP2 was normalized to endogenous *β*-actin mRNA for each sample. (b) The relative protein level of UCP*2* was normalized to endogenous *β*-actin protein for each sample. Dates are expressed as mean ± SD of four individual experiments. **P < 0.01 compared with sham group, *P < 0.05 compared with sham group.

Global cerebral I/R resulted in a significant increase of *UCP2* mRNA expression in the rat hippocampus between 2 h and 15 d after I/R, with the peak expression at 48 h. Meanwhile, the UCP2 protein level in the rat hippocampus significantly increased from 6 h to 15 d, with the peak time of expression at 48 h (Figures 3(a) and 3(b)).

**Figure 4:**
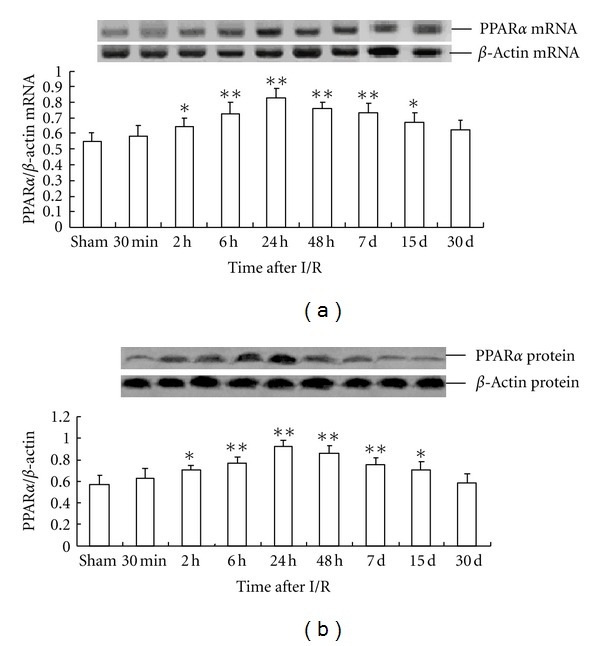
Time-dependent expression of PPAR*α* in global cerebral I/R rat hippocampus (*n* = 4). (a) The relative mRNA level of PPAR*α* was normalized to endogenous *β*-actin mRNA for each sample. (b) The relative protein level of PPAR*α* was normalized to endogenous *β*-actin protein for each sample. Dates are expressed as mean ± SD of four individual experiments. **P < 0.01 compared with sham group; *P < 0.05 compared with sham group.

Expression of *PPAR*α** mRNA in the rat hippocampus increased in the group with reperfusion for 30 min after global cerebral ischemia. However, there was no significant difference between the 30 min reperfusion group and sham operation group. After 2 h of reperfusion after global cerebral ischemia, *PPAR*α** mRNA expression in the rat hippocampus significantly increased compared with that of the sham operation group. At 24 h after I/R, *PPAR*α** mRNA expression reached the peak level, which was about 51% higher than that in the sham operation group (0.83 ± 0.065 versus 0.55 ± 0.053). Thereafter, *PPAR*α** mRNA expression began to decrease. The *PPAR*α** mRNA level at d 30 after I/R was only slightly higher than that in the sham operation group, with no significant difference between the two groups (Figure 4(a)).

The time-dependent course of PPAR*α* protein expression in the rat hippocampus was similar to that of *PPAR*γ** mRNA expression. The peak time of PPAR*α* protein expression was 24 h after I/R, and the peak concentration was about 63% higher than that in the sham operation group (0.93 ± 0.055 versus 0.57 ± 0.091) (Figure 4(b)). 

**Figure 5:**
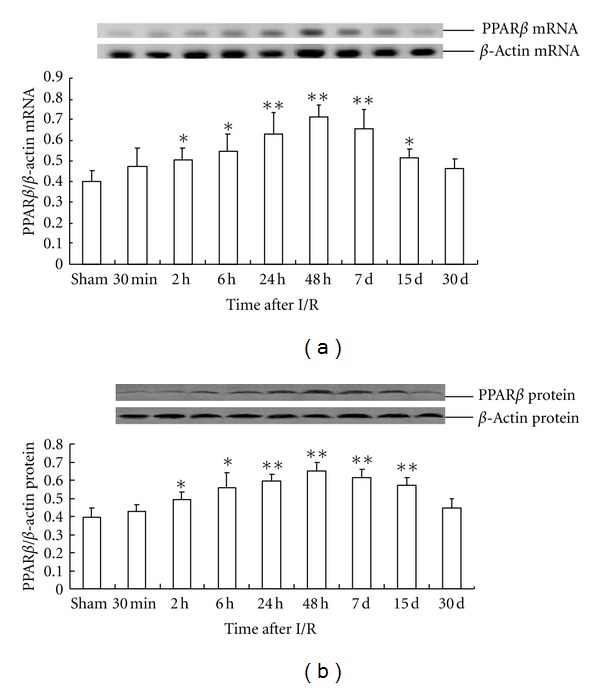
Time-dependent expression of PPAR*β* in global cerebral I/R rat hippocampus (*n* = 4). (a) The relative mRNA level of PPAR*β* was normalized to endogenous *β*-actin mRNA for each sample. (b) The relative protein level of PPAR*β* was normalized to endogenous *β*-actin protein for each sample. Dates are expressed as mean ± SD of four individual experiments. **P < 0.01 compared with sham group; *P < 0.05 compared with sham group.

I/R treatment significantly increased the expression of *PPAR*β** mRNA in the rat hippocampus at 15 days after global cerebral ischemia and reperfusion, with the peak time of expression at 48 h. The level of *PPAR*β** mRNA expression in the 48 h I/R-treated group was about 76% higher than that in the sham operation group (0.71 ± 0.057 versus 0.40 ± 0.05) (Figure 5(a)).

**Figure 6:**
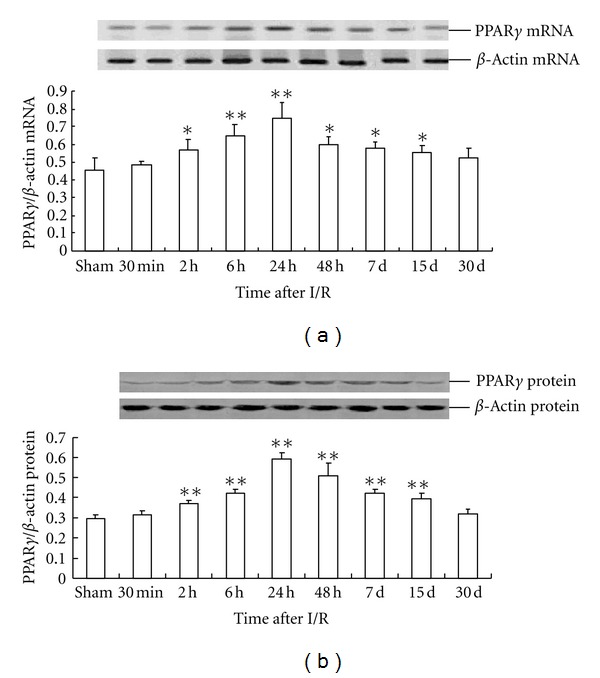
Time-dependent expression of PPAR*γ* in global cerebral I/R rat hippocampus(*n* = 4). (a) The relative mRNA level of PPAR*γ* was normalized to endogenous *β*-actin mRNA for each sample. (b) The relative protein level of PPAR*γ* was normalized to endogenous *β*-actin protein for each sample. Dates are expressed as mean ± SD of four individual experiments. **P < 0.01 compared with sham group; *P < 0.05 compared with sham group.

The pattern of PPAR*β* protein expression was similar to that of *PPAR*β** mRNA expression. The level of PPAR*β* protein expression in the 48 h I/R-treated group was about 65% higher than that in the sham operation group (0.65 ± 0.045 versus 0.39 ± 0.053) (Figure 5(b)). Compared with that of the sham operation group, *PPAR*γ** mRNA expression in the rat hippocampus of the I/R-treated group significantly increased, peaking at 24 h after I/R and then gradually decreased until finally approaching the control level at d 30. The peak concentration of *PPAR*γ** mRNA expression in the I/R-treated group was about 63% higher than that in the sham operation group (0.75 ± 0.085 versus 0.46 ± 0.068) (Figure 6(a)).

The effect of global cerebral I/R on PPAR*γ* protein expression in the rat hippocampus was similar to that of *PPAR*γ** mRNA expression, with the peak time of expression at 24 h after I/R. However, the peak concentration was about 101% higher than that in sham operation group (0.59 ± 0.031 versus 0.29 ± 0.021) (Figure 6(b)).

## 4. Discussion

By using immunohistochemistry and *in situ *hybridization, Braissant and Wahli found that PPARs (PPAR*α*, *β*, and *γ*) show specific time- and tissue-dependent patterns of expression during fetal development in rodents and that the expression of PPARs regulates the development of CNS [21]. Moreno et al. [5] further studied the distribution of PPARs in the adult rat CNS and found that they are expressed in neurons of key brain regions such as the hippocampus and corpus striatum and that the expressed PPARs participate in motor and cognitive functions of the normal CNS and in corresponding dysfunctions in neurodegenerative pathologies. Recent studies showed that agonists of PPARs (PPAR*α*, *β*, and *γ*) are protective against brain injury (i.e., ischemic cerebral damage and traumatic brain injury) and neurodegeneration (i.e., Alzheimer's disease and Parkinson's disease) [2, 22–24]. However, previous studies showed that the protective effects of PPAR agonists depend on the expression levels of the PPARs and their cofactors [19]. Therefore, it is necessary to clarify the time course of expression of PPARs in the cerebral hippocampus subjected to cerebral I/R injury.

In this study, our experimental results showed that the expression levels of PPAR*α*, *β*, and *γ* mRNA and protein in hippocampi were significantly increased during 15 days after global cerebral I/R and peaked at 24 h for PPAR*α* and *γ* and at 48 h for PPAR*β*. Although the time course of PPAR*α*, *β*, and *γ* mRNA and protein expression in the hippocampus has not been reported in either the global or focal cerebral ischemia and reperfusion model, Zhao et al. previously found that in the peri-infarct cortical area of rats, the number of PPAR*γ* immunoreactive cells dramatically increased 12 h after MCAO, then returned to basal values and remained unchanged until the end of the observation period of 7 days [15]. Victor et al. reported that PPAR*γ* expression significantly increased between 4 h and 14 d and peaked at 24 h following MCAO [25]. Another previous study showed a persistent upregulation of PPAR*α*-binding activity and protein expression in the injured human cerebral cortex 6–98 h after trauma brain injury, peaking between 24 and 72 h after injury [26]. 

Much evidence exists suggesting that effective neuroprotective compounds against ischemic cerebral injury require a wide therapeutic time window. In fact, in most cases, it is not clinically possible for drugs to be administered immediately after damage [27]. Considering that the protective effects of PPARs agonists depend on the expression levels of PPARs, our results showed that the peak time for PPAR*α*, *β*, and *γ* expression was 24 h, 48 h, and 24 h, respectively, suggesting that administration of agonists against those molecules before those peak times after ischemia and reperfusion may be effective against ischemic brain injury. Zhao et al. found that treatment with RGZ improves behavioral functions even when first administered 24 h after embolic stroke and suggested that RGZ may potentially widen the therapeutic window of ischemic stroke [15]. Those results also partially support our hypothesis. 

Mitochondrial uncoupling protein 2 (UCP2) is widely distributed in the rat brain including the hippocampus and is regarded as an important player in normal neuronal function as well as a key cell death suppression factor [28, 29]. Kainic acid has been shown to significantly upregulate *UCP2* mRNA expression with the peak level at 24 h and returning to basal levels within 72 h after injection [30]. Lebedev and Arkhipov reported an increase in the level of *UCP2* mRNA in the hippocampus one week after microinjection of kainic acid [31]. SOD2 is one of the most important antioxidative stress proteins. Previous studies have shown that transient global ischemia (TGI) can significantly increase expression of UCP2 and SOD2 in the hippocampal CA1 neurons 4–48 h after TGI [32]. In our study, global cerebral I/R could also significantly increase SOD2 and UCP2 mRNA and protein levels in the rat hippocampal CA1 tissue. Regarding the PPAR signaling pathways, it has been shown that local cerebral ischemic damage does not induce *UCP2 *mRNA in PPAR*β* knockout mice [10]. In other studies, TGI increased UCP2 expression in the mitochondria of hippocampal CA1 subfield 2–24 h after I/R, reaching peak levels at 6–18 h, while preadministration of RGZ to the hippocampus further enhanced mitochondrial UCP2 expression 2–6 h after I/R [33]. These results indicated that there is a PPARs-UCP2/SOD2 neuroprotection cascade in ischemic brain injury. Interestingly, our studies showed that the peak time of SOD2 mRNA is 48 h and the peak time of SOD2 protein is 15 d. The reason of the great time delay was unclear and was necessary to explore.

Moreover, our studies showed that although the expression levels of PPARs, SOD2, and UCP2 in the hippocampus were significantly induced, the rats still displayed obvious impairment of spatial learning and memory and obvious damage to the hippocampal neurons. These observations may be due to the increase of PPARs, SOD2, and UCP2 levels being insufficient to antagonize the damage caused by I/R. Therefore, exogenous PPARs agonists should be supplied. However, considering the fact that the transcriptional activity of PPARs and expression of co-factor LIM-only protein 4 (LMO4) are essential for ligand binding and receptor activation; in future studies, we will observe those levels at different times after cerebral I/R.

In conclusion, our experimental results indicate that global cerebral I/R can induce obvious increases of PPAR*α*, *β*, and *γ* mRNA and protein expression 15 d after I/R, with peak expression times for PPAR*α*, *β*, and *γ* at 24 h, 48 h, and 24 h, respectively. If confirmed, these results may help to guide the experimental and clinical use of PPARs agonists in the future.

## Figures and Tables

**Table 1:** Effect of global cerebral I/R on spatial learning and memory in rats (*n* = 9).

			Exploring time (s)		
	Day 1	Day 2	Day 3	Day 4	Day 5
Sham	112.46 ± 16.14	72.33 ± 15.20	42.48 ± 3.50	23.25 ± 2.30	18.78 ± 2.70
I/R	166.79 ± 23.7	129.05 ± 8.3**	77.53 ± 2.3**	65.73 ± 8.3**	42.48 ± 3.50**

Rats in I/R group were treated with global cerebral ischemia for 20 min, followed by reperfusion for 7 d. Dates are expressed as mean ± SD of four individual experiments. ^*∗∗*^ *P* < 0.01 compared with sham group.
